# Coral reef grazer-benthos dynamics complicated by invasive algae in a small marine reserve

**DOI:** 10.1038/srep43819

**Published:** 2017-03-09

**Authors:** Kostantinos A. Stamoulis, Alan M. Friedlander, Carl G. Meyer, Iria Fernandez-Silva, Robert J. Toonen

**Affiliations:** 1 Curtin University, Perth, Australia; 2 Fisheries Ecology Research Lab, University of Hawai’i at Mānoa, Honolulu, HI, USA; 3 National Geographic Society, Washington, DC, USA; 4 Hawai’i Institute of Marine Biology, University of Hawai’i, Kāne’ohe, HI, USA; 5 California Academy of Sciences, San Francisco, CA, USA.

## Abstract

Blooms of alien invasive marine algae have become common, greatly altering the health and stability of nearshore marine ecosystems. Concurrently, herbivorous fishes have been severely overfished in many locations worldwide, contributing to increases in macroalgal cover. We used a multi-pronged, interdisciplinary approach to test if higher biomass of herbivorous fishes inside a no-take marine reserve makes this area more resistant to invasive algal overgrowth. Over a two year time period, we (1) compared fish biomass and algal cover between two fished and one unfished patch reef in Hawai’i, (2) used acoustic telemetry to determine fidelity of herbivorous fishes to the unfished reef, and (3) used metabarcoding and next-generation sequencing to determine diet composition of herbivorous fishes. Herbivore fish biomass was significantly higher in the marine reserve compared to adjacent fished reefs, whereas invasive algal cover differed by species. Herbivorous fish movements were largely confined to the unfished patch reef where they were captured. Diet analysis indicated that the consumption of invasive algae varied among fish species, with a high prevalence of comparatively rare native algal species. Together these findings demonstrate that the contribution of herbivores to coral reef resilience, via resistance to invasive algae invasion, is complex and species-specific.

Herbivorous fishes and sea urchins are primarily responsible for the high grazing intensity found in healthy coral reef ecosystems^1^,^2^,^3^. This intense grazing pressure has a large influence on the distribution of algae on coral reefs, with macroalgae generally rare in reef zones with the highest grazing pressure^2^,^4^,^5^. Although there has been some debate regarding the mechanisms and causality of competition between algae and corals^6^ high algal biomass has been shown to have a negative effect on coral health^7^,^8^,^9^. Based on their role in algal removal, herbivorous fishes are considered to promote reef resilience and assist in reef recovery to coral dominated states after a disturbance^10^,^11^,^12^.

Over the past several decades, blooms of both indigenous and introduced marine algae have become common on coral reefs, greatly altering the health and stability of nearshore ecosystems^10^,^13^. On reefs subjected to anthropogenic disturbances such as increased terrestrial nutrient inputs or the removal of grazers by overfishing, algal growth rates may exceed grazing rates, resulting in overgrowth of hard corals and other non-mobile benthic invertebrates, and suppression of coral recruitment^14^,^15^,^16^. Herbivorous fishes, particularly large parrotfishes and surgeonfishes, have been severely overfished in many locations/regions and reduction in these grazers has been thought to contribute to increases in macroalgae and subsequent decreases in coral cover^17^,^18^,^19^. Introduced species of macroalgae are often not subject to ecological controls that normally limit abundance in their native range, such as high grazing pressure from native herbivores, allowing them to become invasive^20^,^21^ and accelerate coral-algal phase shifts on coral reefs.

Herbivorous fishes play an important role in promoting resilience and supporting coral recovery, and tend to have small home ranges, suggesting that relatively small-scale (1–10 s km) variation in their abundance will contribute to local changes in rates of reef recovery^22^,^23^. Therefore, local-scale management measures that decrease fishing mortality and increase the abundance and size of herbivorous fishes, such as marine reserves, are expected to play a significant role in supporting recovery and resilience of coral reef ecosystems^24^,^25^. The extent to which different species of herbivorous fishes control macroalgae and promote coral recovery will also depend on their functional role and the algae upon which they graze^26^. Herbivorous fish species tend to be selective in their feeding preferences^27^,^28^ and are typically classified into four functional groups – scrapers, excavators, grazers, and browsers – based on feeding behavior, jaw morphology, and feeding preferences. These groups have different and complementary roles in resisting macroalgae proliferation and promoting coral recovery^29^.

Marine reserves in Hawai’i harbor higher standing stocks of herbivorous fishes and typically have lower macroalgae cover compared with similar adjacent areas^30^,^31^. In Kāne’ohe Bay on the east side of the island of O’ahu, four invasive alga are competitively dominant: *Gracilaria salicornia , Acanthophora spicifera, Kappaphycus alvarezii* (clade A and B)^32^ and *Euchema denticulatum* (clade E)^32^. *G. salicornia , K. alvarezii*, and *E. denticulatum* form extensive blooms and have been observed to invade coral habitat and overgrow reef building corals^33^,^34^. Because *K. alvarezii* (clade A and B) and *E. denticulatum* (clade E) are not easily distinguishable in the field and share the same habitats, they were grouped into a species complex that will be collectively referred to as *Kappaphycus* spp.^35^. Herbivorous fishes in Hawai’i have been found to graze on invasive macroalgae to varying extents and even preferred *A. spicifera* over many native species^21^,^28^. The objective of this study was to determine what effect increased biomass of herbivorous fishes has on invasive algal abundance and distribution. This was achieved by (1) comparing herbivorous fish and invasive algae populations in a marine reserve and adjacent control reefs (Fig. 1); (2) determining movement patterns of herbivorous reef fishes among reefs in the study area using acoustic telemetry; and 3) quantifying the dietary contribution of alien and native algal species among herbivorous reef fishes in the study region using a genetic approach based on metabarcoding and next-generation DNA sequencing.

## Results

### Herbivore biomass and size inside vs. outside of marine reserve

Spatial autocorrelation was tested and the spatial distribution of herbivorous fish biomass among sample locations was found to be random (z = 1.6, p = 0.6). The biomass of herbivorous fishes differed significantly among habitat types (F_1,2_ = 178.6, p < 0.0001) and was nearly three times higher inside the marine reserve (33.0 ± 6.6 s.e.m. g m^−2^) compared to fished reefs (13.4 ± 2.2 s.e.m g m^−2^) for all habitats combined (F_1,2_ = 20.9, p < 0.0001, Fig. 2). For both protected and fished reefs, herbivore biomass was lowest on the reef flat and did not differ significantly between slope and crest habitats (Tukey’s HSD: reserve p = 1.0, open p = 0.4). Abundance size spectra of herbivorous fishes in the reserve and open area (Fig. 3) showed a significant difference in midpoint height (F_1,1_ = 5.2, p = 0.046) though not for slope (F_1,1_ = 0.8, p = 0.4). This indicates a higher abundance of fishes across all size classes >10 cm in the reserve compared to the open areas. Despite the difference in overall biomass, herbivore assemblage structure was nearly identical between management zones. The same five parrotfish and eleven surgeonfish species representing grazers, scrapers, and browsers were recorded in both areas with very similar rankings in terms of relative mean biomass (Supplementary Table S1).

### Herbivore movements

A total of 40 individual herbivorous fishes from four species were acoustically tagged in the reserve. These included two surgeonfishes (*Acanthurus xanthopterus, Naso unicornis*) and two parrotfishes (*Calotomus carolinus, Chlorurus perspicillatus*). Fifteen individuals from three of these species were detected for more than a year (Supplementary Table S2). These herbivorous fishes resided predominantly inside the reserve, with only 1.3% of total detections occurring on the control reefs (Fig. 4). An average of 92% of the days detected were on receivers inside the marine reserve (Supplementary Table S2). Eleven individuals had nearly 100% of detection days inside the reserve, three fishes had between 75–78% of detection days inside the reserve, and one individual (*N. unicornis*, 25.6 cm) had 56% of detection days inside the marine reserve. This single fish showed a different pattern than all others, coming into the reserve to feed during the day and sheltering on an adjacent reef at night.

### Benthic community and invasive algae cover

Spatial autocorrelation was tested and the spatial distribution of coral cover (z = 0.5, p = 0.6), total macroalgae cover (z = −1.8, p = 0.07), and invasive algae cover (z = 1.0, p = 0.3) among sample locations were found to be random. Benthic community composition was consistent between fished and unfished reefs as indicated by an analysis of similarities (ANOSIM) of substrate type percent cover (R = −0.3, p = 0.8). However, the difference in benthic community composition between habitats was highly significant (R = 0.8, p = 0.001). The reef crest was dominated by coral, crustose coralline algae, and turf; the reef flat consisted of soft substrate, primarily sand and rubble, with macroalgae being the dominant biotic cover. The reef slope had the highest cover of coral and the lowest macroalgae cover (Table 1). Across all habitats, coral cover was significantly higher in the open area (F_1,2_ = 4.1, p = 0.046). There was no difference in total macroalgae cover between management types (F_1,2_ = 0.01, p = 0.9), though native macroalgae, as a group, was significantly higher in the open area (F_1,2_ = 4.8, p = 0.03). As a group, invasive algae cover showed no significant effect of management (z = −1.4, p = 0.2) or herbivore biomass (z = −1.4, p = 0.2). However, at the species level, invasive algal cover showed significant, but differing, results between management zones. *A. spicifera* was more abundant in the open areas (z = 2.8, p = 0.005) and was negatively associated with herbivore biomass (z = −2.8, p = 0.005), while *G. salicornia* showed higher abundance in the marine reserve (z = −3.8, p = 0.0001) and had no association with herbivore biomass (z = −1.5, p = 0.1). *Kappaphycus* spp. showed no effect of management (z = 1.1, p = 0.3) or herbivore biomass (z = 0.1, p = 1.0). *A. spicifera* was exclusively found on sandy reef flats. *G. salicornia* was predominantly observed on reef flats while *Kappaphycus* spp. were found primarily on the hard bottom reef crest, though not in slope habitats (Fig. 5). In comparison, native macroalgae were generally found on the reef crest and to a lesser extent on the reef flat. Thus, total macroalgae cover was close to 10% for both the reef crest and reef flat habitats (across management strata), with the sandy flats dominated by invasive species and the reef crest composed primarily of native species (Table 1, Fig. 5).

### Herbivore diets

After removing false positives (OTUs matching records of species not known to be present in Hawai’i) and merging redundant genetic markers, we identified 34 distinct species or species groupings of macroalgae in the gastrointestinal samples and one class of planktonic green algae (Supplementary Table S3). Based on these results, the herbivorous fishes examined in this study fed predominately on the native *Asparagopsis taxiformis,* followed by the invasive *G. salicornia*. These two algal species made up an average of nearly 50% of algal taxa OTUs identified across all fish species sampled (Table 2, Fig. 6). Interestingly, unicellular green algae of the class *Prasinophyceae* were present in gastrointestinal samples from all species and made up 18% of identifiable algal taxa on average (Fig. 6, Table 2). The other invasive algae *Kappaphycus* spp. and *A. spicifera* made up an average of 7% and 2% of identifiable algal samples, respectively, with *Kappaphycus* spp. consumed by five out of the seven species examined and *A. spicifera* consumed by three (Table 2, Fig. 6).

Of the herbivorous fish species tested, the convict tang (*Acanthurus triostegus*) consumed the largest number of algal species identified in the samples (31), including the invasive alga *G. salicornia* and *A. spicifera*. The two parrotfishes examined (*Calotomus carolinus* and *Chlorurus perspicillatus*) were found to consume the least number of identified algal species, five and eight respectively, though these included the invasive *G. salicornia* and *Kappaphycus* spp. (Supplementary Table S3, Fig. 6). The blue-spine unicornfish (*N. unicornis*) and spectacled parrotfish (*C. perspicillatus*) were the only species tested that had all three invasive algal species present in their gastrointestinal tracts (Fig. 6).

## Discussion

If herbivore grazing suppresses macroalgal proliferation, then benthic cover of macroalgae should be inversely related to herbivore biomass among habitats with similar environmental characteristics. We found this pattern for native macroalgae, though results for invasive macroalgae were equivocal and species specific. The invasive macroalgae examined in this study represent the dominant algae in Kāne’ohe Bay^33^,^34^. Among these, *G. salicornia* and *Kappaphycus* spp., form extensive blooms and have been observed to invade coral habitat and overgrow reef building corals in Hawai’i^33^,^34^. *A. spicifera* was found primarily on the sandy reef flat and therefore does not directly overgrow coral reefs. *Kappaphycus* spp. were found only on hard-bottom reef crests where they can overgrow live coral, whereas *G. salicornia* was found in all habitats, but achieved its highest abundance on reef flats.

Phase shifts can occur when rates of consumption by herbivores fail to equal algal growth rates^36^,^37^,^38^. Grazing rates differ among habitats in Kāne’ohe Bay with the highest grazing pressure occurring in the reef slope and crest habitats near the edge of patch reefs and decreases with distance towards the center of the reef flat^28^, likely due to shelter from predation provided by these structurally complex habitats^39^,^40^. Lower grazing pressure in reef flat habitats could create refugia for algae and partly explain why some algal species remain prevalent inside the marine reserve despite the presence of abundant herbivores in adjacent habitats^41^. Furthermore, our diet analysis showed different combinations and proportions of macroalgal species consumed among the herbivorous fishes sampled. As a result, ecological responses of invasive macroalgae to grazing by herbivorous fishes varied by species and habitat, corroborating previous work^21^,^28^,^34^.

The seven species of herbivorous fishes whose diets were examined in this study (with the exception of *Calotomus carolinus*) were among the most abundant observed in the marine reserve and include the endemic parrotfish *Chlorurus perspicillatus* (Supplementary Table S1). They represent all the herbivore functional groups defined by Green and Bellwood (2009) except for bioeroders/large excavators (though *Chlorurus* spp. ≥35 cm are considered bioeroders, all the specimens we examined were below this size) and their diets differed widely in terms of species of macroalgae consumed. Although the approach is not strictly quantitative, qualitative conclusions can be inferred about the grazing pressure of different fishes on different algal species. *G. salicornia* was the invasive macroalgae consumed most commonly, whereas *Kappaphycus* spp. and *A. spicifera* were among the least commonly detected in the diets of herbivorous fishes (Table 2). The relative consumption of invasive algal species corresponded to their prevalence in the benthic surveys in the marine reserve (Table 2, Fig. 6), particularly crest and slope habitats where the majority of herbivorous fishes were found (Fig. 2). Stimson *et al*.^39^ and Conklin^28^ conducted herbivore preference tests comparing introduced and native macroalgae in Kāne’ohe Bay, and found *A. spicifera* to be the most preferred and *G. salicornia* and *Kappaphycus* spp. to have intermediate to low preference. These results therefore suggest that algal abundance in preferred habitats plays a greater role in diet selection than herbivore preferences.

The most frequently-detected alga found in the herbivorous fish species diets was the native *A. taxiformis*. This algal species was recorded on our benthic surveys, though only in the open area during the winter months. It’s not surprising that we did not detect it in the reserve given its high prevalence in fish diets combined with high herbivore biomass. Furthermore, it’s likely that the herbivorous fishes tested were feeding on the Falkenbergia phase of *A. taxiformis*^42^ which would be present in the turf and therefore not identifiable by our methods.

When collecting fecal samples from one of the six herbivore species that appear to commonly consume *G. salicornia* (*N. unicornis*), we noticed that for many individuals the feces contained visibly identifiable, undigested fragments of *G. salicornia*. Bierwagen *et al*.^43^ tested the hypothesis that a portion of these egested fragments was viable and found a 17% survival rate, indicating that these fish can potentially disperse *G. salicornia*. Vermeij *et al*.^44^ describes the same process for herbivorous fishes in the Caribbean where they found a 76% survival rate for algal fragments belonging to the group Rhodophyta, the same division to which *G. salicornia* belongs. Furthermore, they observed that while herbivorous fishes in their study fed predominately on hard strata, they defecated primarily on sand, a behavior also known from other herbivorous fish species^45^,^46^,^47^. Thus, we must consider the likelihood that *N. unicornis*, one of the largest and most abundant herbivores observed in the marine reserve, may be dispersing *G. salicornia* locally on the sandy reef flat, at least partially offseting its grazing impact on this species.

This study suggests that a small (0.3 km^2^) no-take marine reserve could be effective at protecting herbivorous fish populations, with average biomass nearly three times higher than adjacent control reefs. These findings are supported by Friedlander *et al*.^30^ who found that total biomass was nearly two times higher inside this reserve compared to fished reefs in Kāne’ohe Bay. Our size spectra analysis indicated that herbivorous fishes in the reserve were more abundant in all size classes >10 cm, which could suggest that reduced fishing has increased overall biomass^48^. The fact that the slopes did not differ likely means the fished reefs experience ‘size-neutral exploitation’^49^ where many size classes are fished in similar proportions. This makes sense given that gill and surround nets account for the majority of the fish catch in Kāne’ohe Bay^50^.

Site fidelity of herbivorous fishes was surprisingly high, with < 7% utilizing adjacent control reefs. This may be partly explained because this marine reserve is a patch reef surrounded by relatively deep water (>10 m), which forms a natural boundary that reef fishes seldom cross^51^,^52^.

Given the lack of replicate reserve and control reefs, we cannot conclude that protection alone is responsible for the observed differences in herbivorous fish biomass. One contributing factor could be that the marine reserve is a fringing reef that has been heavily altered by dredging, which has produced channels/inlets, spits, and a number of moats. It is possible that these features could be responsible for some of the observed characteristics of the fish fauna by providing new habitats not found on the control reefs. Nevertheless, the results of this study, in terms of overall herbivorous fish biomass, size distribution, and site fidelity, provide a case that this small reserve, with natural habitat boundaries to emigration, could potentially be effective in protecting the localized herbivorous fish assemblage^52^,^53^,^54^.

Many researchers have shown benefits of, and advocated for, the use of marine reserves to support resilience of coral reefs through protection and enhancement of herbivore populations^3^,^10^,^55^,^56^. How these populations influence resilience in the presence of invasive macroalgae is not as well understood. Introduced macroalgae can have advantages over indigenous species that allow them to become invasive due to relaxation of external regulatory and limiting processes that normally control their abundance in the native range^21^. In this study we found two non-indigenous, invasive, macroalgal species dominated the reef flat and slope habitats relative to native algae (Fig. 5), and for which consumption by herbivores varied widely (Fig. 6). Between these, *A. spicifera* is most preferred by herbivores fishes^28^, was significantly more abundant in the open area compared to the reserve, and was negatively associated with herbivorous fish biomass. Thus its distribution is influenced by the abundance of herbivorous fishes and marine protection could be an effective control for this species. Though our diet analysis shows *A. spicifera* to be consumed by only 3 of 7 herbivorous fish species tested, this could reflect the fact that these fishes were all collected within the marine reserve where prevalence of this alga is low.

In contrast, *G. salicornia* was the dominant macroalgal species in the marine reserve, and appeared to thrive despite the presence of a large and healthy herbivorous fish population. We believe this is due to its predominance in habitats with low grazing pressure (sandy reef flats), competitive release from the more highly palatable *A. spicifera*, and fecal dispersal of viable fragments by *N. unicornis* and potentially other species. Furthermore, we cannot rule out the potential influence of terrestrial nutrients from the developed and populated island located in the middle of the reserve reef. Another contributing factor may be suppression of herbivory by the high density of established *G. salicornia* patches. Hoey and Bellwood^57^ found that both grazing and browsing fishes avoided high density macroalgae patches, preferring relatively open areas with low macroalgal cover. This behavior may provide a positive feedback by promoting the growth and persistence of *G. salicornia* in the marine reserve. Conklin^28^ experimentally cleared areas on the leeward reef slope of the reserve of *G. salicornia*, showing that algae cover remained low and was declining well after removal efforts had ceased, which likely resulted from macroalgal abundance being reduced to levels where herbivores were able to control further growth^28^. In contrast, algal removal in areas with depressed herbivore populations (including unprotected patch reefs in Kāne’ohe Bay) show rapid regrowth of *G. salicornia* following experimental removal which has led to efforts to stock urchins following removal in areas open to fishing^58^. A next logical step would be to reproduce Conklin’s^28^ experimental removal of *G. salicornia* in the marine reserve at a larger scale, in order to determine if the healthy herbivore populations can control this alga at low levels of abundance, or if the ecological feedbacks discussed above would eventually restore it to dominant levels.

*Kappaphycus* spp. have intermediate preference^28^ were consumed by most of the herbivores we tested, and were primarily restricted to habitats where grazing pressure is highest (reef crest)^28^. While abundant elsewhere in Kāne’ohe Bay^35^ it formed a high proportion of cover (12–21%) at only a few sites at the northernmost edge of control reef one and was present at low levels at several sites on the leeward (W) shore of the reserve. Likely due to this patchy distribution, cover of *Kappaphycus* spp. did not differ statistically between the fished and reserve reefs and was not linked to herbivore biomass. This species is of particular concern due to its competitive dominance, large size, and ability to overgrow live coral^34^. The State of Hawai’i and The Nature Conservancy have worked to manage *Kappaphycus* spp. elsewhere in Kāne’ohe Bay via mechanical removal and sea urchin bio-control^58^. Based on the results of our diet analysis, we propose that protection of herbivores may also be a beneficial tool for management. In particular, the large bodied, native herbivore *Naso unicornis* for which *Kappaphycus* spp. makes up the greatest proportion of its diet out of all species tested (Fig. 6).

The most often used metric of reef health is coral cover, though Hughes *et al*.^59^ note that it is not a reliable metric of resilience. Nevertheless, high coral cover is associated with healthy herbivore populations^25^,^60^. thus it is counterintuitive that our data shows both significantly lower herbivore biomass and higher coral cover on fished reefs compared to the marine reserve. This is likely due to the fact that this reserve surrounds a highly modified and populated island, thus lower coral cover may be a result of indirect human impacts such as from terrestrial runoff 61. This highlights the potential importance that indirect impacts from human activities have on structuring coral reef communities and further investigation is warranted. Ratios of coral to macroalgae cover (~2–3) in the reef crest habitats we examined where both are prevalent show that this system appears to not currently be at risk of a phase shift^59^ and cover of macroalgae in these habitats are at the low end of the range found in the Indo-Pacific (9–12%) as described by Bruno *et al*.^62^. Nevertheless, two species of invasive macroalgae form a large proportion of this cover, making these habitats potentially vulnerable in the event of a large scale disturbance such as widespread coral bleaching especially where herbivores are not protected^17^,^36^.

Marine protection has been shown to promote resilience of coral reefs^3^,^56^. However, the introduction of non-indigenous, highly competitive, invasive macroalgae can complicate or undermine ecological relationships established through co-evolution and increase the likelihood of phase shifts. This is especially true in the face of anthropogenic impacts and climate change which promote macroalgal growth. This study revealed varying degrees of diet specialization among herbivores, suggesting that functional redundancy among this group may be lower than previously thought^27^. In addition, ecological feedbacks such as algal dispersal by herbivores and variable grazing pressure based on habitat and density of established algal patches may combine to promote the persistence of macroalgae, especially invasive species. Thus, while no-take marine reserves can support resilience of coral reefs through protection of herbivores, they are not a panacea, and additional measures such as wider protection for critical species such as *Naso unicornis*, and managing terrestrial runoff and nutrient inputs, may be necessary when confronted with unpredictable factors such as invasive algae. Care must be taken to understand species-specific differences, in both herbivores and macroalgae, in order to inform targeted and effective management for coral reef resilience.

## Methods

### Study sites and habitats

**Figure 1:**
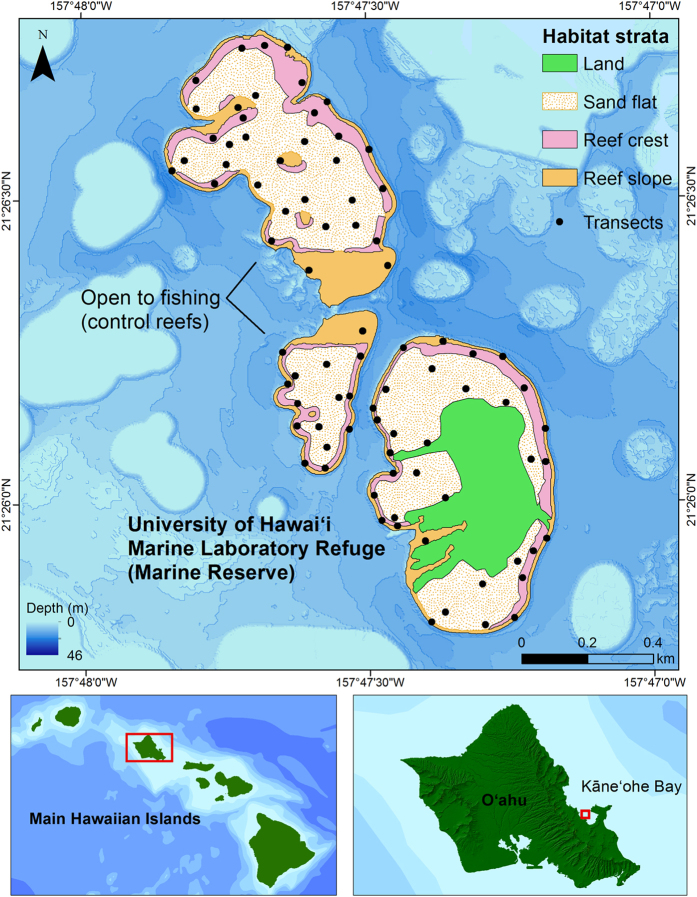
Study area in the south end of Kāne’ohe Bay showing reserve and control reefs as well as habitat strata and transects. Map was created using ESRI ArcMap 10.1, http://www.esri.com/software/arcgis/arcgis-for-desktop.

**Figure 2:**
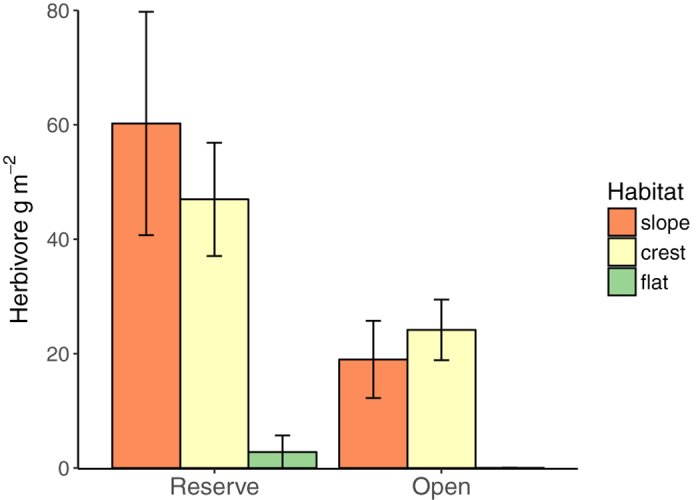
Mean herbivorous fish biomass (gm^−2^) across habitats and management types. Error bars represent standard error of the mean (s.e.m.).

**Figure 3:**
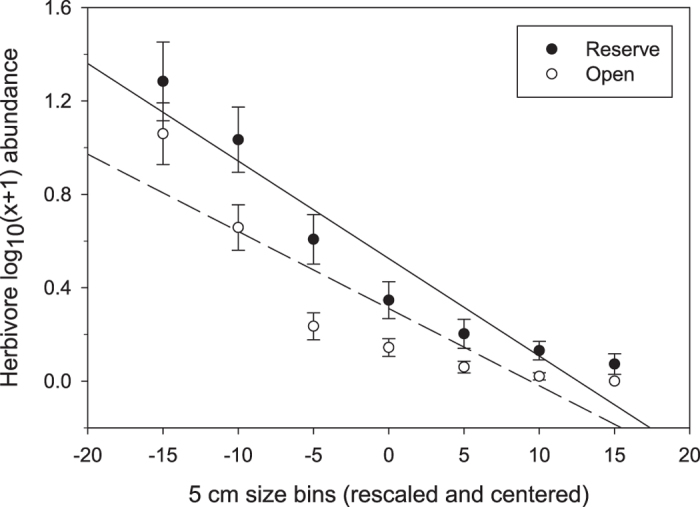
Size spectra analysis of herbivorous fishes. Mean log_10_(x + 1) transformed abundance by 5 cm size classes for reserve and open areas, rescaled so the midpoint is zero and fitted with ordinary least squares linear regressions (solid line is reserve, dashed line is open area).

**Figure 4:**
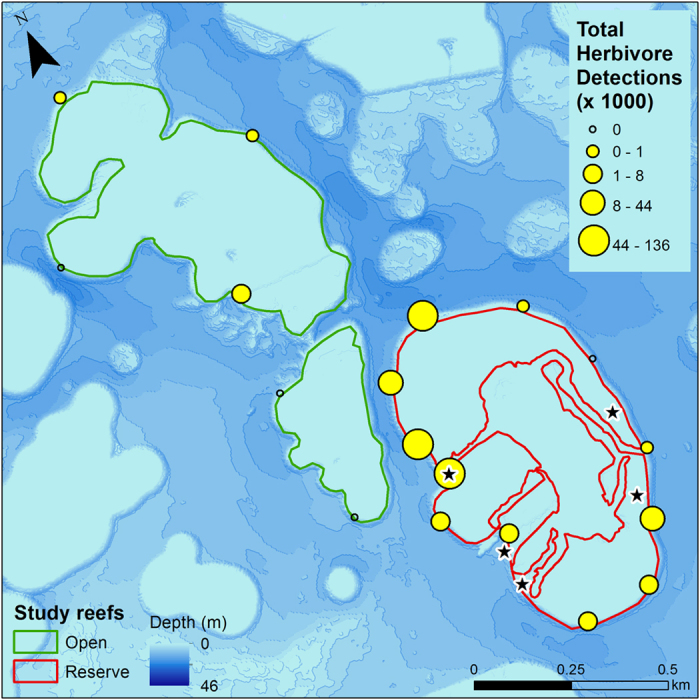
Total detections of tagged herbivorous fish for each receiver. All fish were captured in the marine reserve. Stars denote capture/release locations for tagged individuals (N = 40). Map was created using ESRI ArcMap 10.1, http://www.esri.com/software/arcgis/arcgis-for-desktop.

This research took place in Kāne’ohe Bay on the windward side of the island of O’ahu (Fig. 1). The University of Hawai’i Marine Laboratory Refuge is a highly modified island surrounded by a 26 ha no-take marine reserve established in 1967 at the south end of the bay. Active surveillance of the reserve occurs 24 hours a day, 5 days a week, though not on weekends. Kāne’ohe Bay contains numerous patch reefs that can be treated as multiple discreet sampling units, allowing us to compare the efficacy of this marine reserve in controlling invasive algae relative to similar patch reefs that lack protection from fishing. Control reefs were chosen based on their proximity to the reserve and similarity in both size and habitat, though they do not contain islands. The area of the protected reef is 26 ha, control reef one (to the north) is 29 ha, and control reef two is 8 ha (Fig. 1). Existing benthic habitat maps^63^ had a minimum mapping unit of 0.4 ha and were not detailed enough for the purposes of this research. Therefore, a new habitat map was created for the study area. Dominant habitat types are the reef crest, which forms the margins of each patch reef and is composed of pavement and aggregate reef, the reef flat (scattered reef, rubble, and sand), and reef slope (coral dominated). The habitat map was generated using a supervised classification method in ArcGIS 10 where habitat polygons were digitized at a 1/2000 scale (Fig. 1).

### Ecological surveys (fishes and benthos)

Ecological surveys were conducted using a stratified random design in which random points were assigned to dominant habitat types. Sample size optimization was based upon data collected by Friedlander *et al*.^30^,^31^ using the number of species and number of individuals per transect among the three major habitat types surveyed in the study area. Based on these results, 9–10 samples per habitat were sufficient to achieve reasonable precision of mean abundance and richness. We allocated 14 transects for the reef flat habitat in the reserve (which encompasses the greatest area) and 10 transects each to the reef crest and slope habitats. Transects were assigned to control reefs based on the same transect to area ratios (flat-17, crest-19, slope-13), so that each habitat type was sampled proportionately, resulting in a total of 83 spatially independent sample locations (Fig. 1). Though transects were placed randomly, we ensured that each transect was spaced at least 60 m from adjacent transects to prevent any overlap. While transect depths varied with tide level, those located on the crest and flat averaged 0.6 m and 0.7 m and were surveyed with snorkel, while the slope transects averaged 4.7 m and were surveyed using SCUBA. All transects were surveyed bi-annually during the winter and summer seasons for a period of two years (2012–2013) to account for seasonal variability in invasive algae growth (DAR unpublished data).

Fish assemblages were quantified using standard underwater visual belt transect survey methods. A diver swam a 25 × 5 m transect at a constant speed and identified to the lowest possible taxon all fishes visible within 2.5 m to either side of the centerline (125 m^2^ transect area). Total length (TL) of fishes were visually estimated to the nearest cm. Length estimates of fishes from censuses were converted to weight using the allometric growth equation: W = *a*(TL)^*b*^ where the parameters *a* and *b* are species-specific constants, TL is total length in cm, and W is weight in g. Fish taxa were categorized into four trophic guilds (herbivores, benthic secondary consumers, planktivores, and piscivores), though only herbivores were considered in this study.

In order to quantify benthic cover, a 0.25 m^2^ quadrat-based point-intercept method was used. Upper canopy benthic cover was assessed along the same 25 m transects as the fish surveys, with each transect stratified into 5 m segments and two quadrats randomly allocated within each segment. Each 0.25 m quadrat contained 16 line intersections under which the substrate type was identified to the lowest possible taxonomic level, resulting in 160 points per transect^31^.

### Herbivore movements

To determine site fidelity of herbivorous fishes to the unfished reef, we deployed 12 acoustic receivers at regular intervals around the marine reserve. Six additional receivers were strategically placed on the control reefs to avoid overlap in detection ranges for receivers inside and outside the reserve. Methods of capture and surgeries of reef fishes for the purpose of this study were approved by the Institutional Animal Care and Use Committee (IACUC) under the University of Hawaii System Office of Research Compliance, and occurred under IACUC protocol #13–1600. These methods were performed in accordance with the relevant IACUC guidelines including appropriate surgical training, anesthetic monitoring, and aseptic procedures. Divers captured herbivorous reef fishes underwater at night by illuminating them with a flashlight and trapping them in soft-meshed hand nets. Captured fishes were transferred to soft mesh bags floating at the surface for subsequent processing. All reef fishes were captured at various locations within the marine reserve. At the end of each capture session, fishes were measured (FL and TL in mm), a fecal sample was obtained, and fishes were held overnight in land-based flow-through 160 l seawater tanks. The following day, fishes were placed into an aerated anesthetic bath (MS 222, 0.15 g l^−1^). Anesthetized fishes were then transferred to a padded surface where a VEMCO V9 acoustic transmitter (9 mm × 21 mm, 1.6 g) was surgically implanted into the body cavity of each fish through a small incision in the abdominal wall (e.g. Meyer and Honebrink^64^). We then externally tagged each fish with a serially numbered, 8.0 cm plastic dart identification tag (Hallprint, South Australia). Tagged fishes were resuscitated in pure seawater and transferred back to the 160 l holding tank. Fishes were examined the following morning, and then released at the location of capture. All tagged fishes were alert and orientating correctly, and swam away vigorously on release.

### Herbivore diets

**Figure 5:**
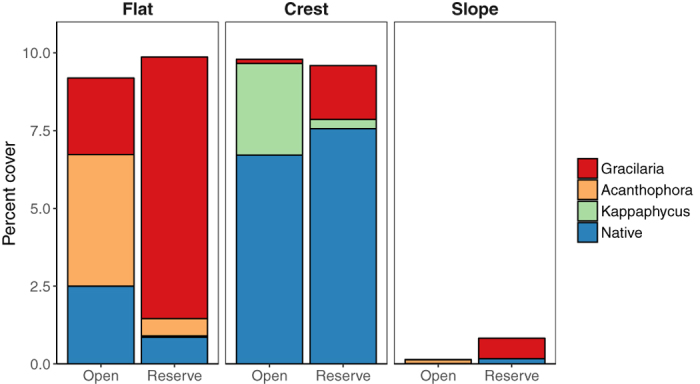
Mean percent cover of invasive and native algae by habitat and management.

**Figure 6:**
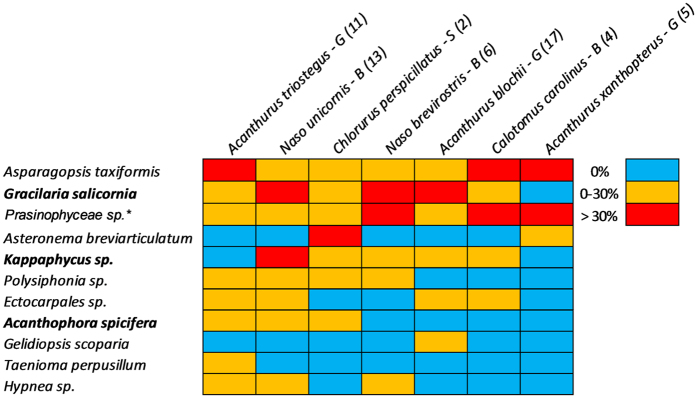
Heat map showing relative proportion of algae species found in herbivore gastrointestinal samples which accounted for at least 5% of identifiable gut contents for any fish species. Sample size (N) for each species provided in parentheses. Values are based on proportion of total OTUs for each herbivore species sampled: absent (0%), present (0–30%), and common (>30%). Each fish species is further identified by herbivore functional group: scraper (S), grazer (G), and browser (B). Invasive algae species are shown in bold. All algal species are macroalgae with the exception of *Prasinophyceae* spp. which is a class of unicellular green algae.

Feces were obtained from specimens of herbivorous fishes collected from the marine reserve from November 2011 to January 2012. In most cases fecal samples were taken from the same specimens implanted with acoustic tags by gently squeezing the ventral surface forward of the anus. In cases where fishes were too small, or of the wrong species for tag implantation, both feces and stomach contents were collected surgically and preserved in 99% ethanol. If feces could not be obtained via gentle squeezing, a sample was obtained from the distal section of the digestive system, as close as possible to the anus. We collected samples for 62 specimens of 5 surgeonfishes and 2 parrotfishes, sample sizes for each species are shown in Fig. 5.

For DNA extraction we disrupted the frozen samples with a mortar in liquid nitrogen and followed the Qiagen Plant DNeasy extraction protocol as described by the manufacturer (Qiagen Venlo, NL). We quantified the DNA using an AccuClear Ultra High Sensitivity dsDNA Quantitation protocol (Biotium Inc., Hayward, CA) and pooled the DNA extracts of samples collected from the same fish species at equimolar concentration. To characterize the algae present in the gastrointestinal samples we used a novel approach that combines metabarcoding with mass-parallel next-generation sequencing (NGS). Our method is similar to that of Leray *et al*.^65^, but, did not require the use of PCR blocking primers because we applied a barcoding gene from the algal plastid genome not present in animals. This process is detailed in the supplementary information. We compiled a list of 23S sequences from algae publicly available in GenBank (www.ncbi.nlm.nih.gov/genbank/) and the Barcode of Life repositories (www.boldsystems.org/index.php/databases) and used it as reference to build seeds and cluster sequences into Operational Taxonomic Units (OTUs) using the uclust_ref algorithm implemented in CHIME^66^ (chime.org). OTUs that matched 23S sequences in the database (using the BLAST method implemented in CHIME) were assigned a taxonomic classification. In order to account for differing sample sizes and OTU counts between fish species, we calculated the proportion of total OTUs per alga by fish species.

The number of OTUs should be roughly proportional to the amount of DNA and thus the number of cells of each algal species in the gastrointestinal samples. However, this relationship has not been validated and many factors can distort it. Furthermore, this method can only detect species for which there are existing 23S sequences in the genetic reference databases. For these reasons, while we report number of OTUs, our analysis and interpretation rest primarily on the identification of algal species for which genetic barcoding information is available, in the herbivorous fish species diets tested.

### Statistical Analyses

Temporal replicates (N = 331) for fish and benthic data were averaged over the four survey rounds and subsequent tests were performed at the transect level (N = 83). An alpha level of 0.05 was used for all statistical tests. Spatial autocorrelation among transects for all response variables was tested using Morans I to confirm sample independence. The effects of management and habitat on herbivorous fish biomass were evaluated using a two-way ANOVA followed by Tukey’s honestly significantly difference (HSD) multiple comparisons test^67^. Data were ln(x + 1) transformed prior to statistical analysis to meet the assumptions of the ANOVA. Normality was tested using a Shapiro-Wilk W test while a Bartlett’s test was used to examine homogeneity of variance. To test herbivore size-related differences between management regimes, we developed size spectra for each management strata. All herbivorous fishes were assigned to one of seven 5 cm size classes from 10–45 cm. Individuals <10 cm were excluded as their abundance is not well represented by underwater surveys^68^ and size classes at the upper end of the range were excluded because they were based on sightings of only one or two individuals^69^. Linear least-squares regressions fitted to the size distributions were based on mean abundance for each 5 cm TL size class. Abundance data was log_10_(x + 1) transformed before analysis to ensure a linear relationship, and the midpoint of size classes was rescaled to the size range and set to zero, giving a value for midpoint height as opposed to intercept, thus removing the correlation between slope and intercept^69^,^70^. The midpoint height is measure of overall assemblage biomass^71^,^72^. Analysis of covariance (ANCOVA) was conducted between the two regression models to test differences between the slopes and midpoint heights^67^.

Benthic community composition between fished and protected reefs was compared with a multivariate analysis of similarities (ANOSIM, PRIMER v6)^73^. The data matrix consisted of percent cover of major benthic cover types between management strata. Cover of benthic functional groups (e.g. coral, macroalgae) were arcsin square-root transformed to meet the assumptions of normality and homoscedasticity and the effects of management and habitat were evaluated using a two-way ANOVA^67^. Invasive algae cover data did not conform to assumptions of normality despite transformation, so zero-inflated, negative binomial generalized linear mixed-effects models (GLMMs) were fit with management and herbivore biomass as the main effects and habitat as a random effect^74^. GLMMs were applied to all three invasive species combined and to each species individually in order to evaluate the effects of management and herbivore biomass, while controlling for habitat.

## Additional Information

**How to cite this article**: Stamoulis, K. A. *et al*. Coral reef grazer-benthos dynamics complicated by invasive algae in a small marine reserve. *Sci. Rep.* **7**, 43819; doi: 10.1038/srep43819 (2017).

**Publisher's note:** Springer Nature remains neutral with regard to jurisdictional claims in published maps and institutional affiliations.

## Supplementary Material

Supplementary Information

## Figures and Tables

**Table 1:** Mean percent benthic cover between management strata and among habitat types.

Management	Habitat	Coral	Macroalgae	Coralline algae	Turf algae	Uncolonized
Reserve	Crest	20.0 (11.5)	9.6 (8.0)	19.5 (17.0)	34.3 (16.1)	15.1 (14.9)
	Slope	34.6 (14.8)	0.8 (3.7)	2.6 (4.3)	10.0 (10.7)	47.9 (20.5)
	Flat	0.5 (1.9)	9.9 (9.8)	0 (0)	1.2 (1.7)	88.1 (9.9)
Open	Crest	30.7 (15.4)	9.8 (7.7)	23.7 (14.9)	24.2 (17.4)	7.9 (9.6)
	Slope	41.1 (24.6)	0.1 (0.9)	1.6 (2.7)	10.0 (8.1)	43.5 (24.0)
	Flat	0.6 (3.0)	9.2 (10.5)	0.1 (0.4)	3.1 (4.6)	86.8 (12.5)

Macroalgae represents total macroalgae (natives and invasives). Standard deviation (s.d.) provided in parenthesis.

**Table 2:** Comparison of field measured cover and prevalence in fish diets of the top 10 macroalgal species observed in the marine reserve.

FIELD	% macro-algae cover	DIET	% fish diets
***Gracilaria salicornia***	57.6 (0.1)	*Asparagopsis taxiformis*	26
Dictyosphaeria versluysii*	28.0 (3.8)	***Gracilaria salicornia***	22
***Acanthophora spicifera***	3.2 (1.3)	*Prasinophyceae* spp.	18
*Sphacelaria* spp.	2.9 (0.1)	*Asteronema breviarticulatum*	12
Lyngbya majuscula*	2.7 (0.6)	***Kappaphycus*****spp.**	7
Halophila hawaiiensis*	1.5 (0.2)	*Polysiphonia* spp.	3
***Kappaphycus*****spp.**	1.5 (12.6)	*Ectocarpales* spp.	3
*Ceramium* sp.	1.2 (1.4)	***Acanthophora spicifera***	2
*Dictyota* sp.	0.4 (0.5)	*Gelidiopsis scoparia*	2
Dictyosphaeria cavernosa*	0.3 (7.8)	*Taenioma perpusillum*	1

Benthic cover in the field is expressed as the percentage of total average macroalgae cover (s.d. in parenthesis) by species. Prevalence in fish diets is represented by the mean percentage of OTUs per algal species by fish species. All algal species represent macroalgae with the exception of *Prasinophyceae* spp. which is a class of unicellular green algae.

^*^Denote species that were not represented in the reference database and therefore could not be identified in the gastrointestinal samples.
