# Supplementary material for: Coral reef grazer-benthos dynamics complicated by invasive algae in a small marine reserve
Source: Sci Rep. 2017 Mar 9;7:43819. doi: 10.1038/srep43819 (PMC5343440; doi:10.1038/srep43819)
Supplement: Supplementary Information [file srep43819-s1.pdf]

Coral reef grazer-benthos dynamics complicated by invasive algae in a small marine reserve

Authors: Kostantinos A. Stamoulis*[1,2], Alan M. Friedlander[2,3], Carl G. Meyer[4], Iria Fernandez-Silva[4,5], Robert J. Toonen[4]

*Correspondance to kostanti@hawaii.edu

1. Curtin University, Perth, Australia;

2. Fisheries Ecology Research Lab, University of Hawaiʻi at Mānoa, Honolulu, HI, USA;

3. National Geographic Society, Washington, DC, USA;

4. Hawaiʻi Institute of Marine Biology, University of Hawaiʻi, Kāneʻohe, HI, USA;

5. California Academy of Sciences, San Francisco, CA, USA

Supplemental Information

Supplementary Tables

Supplementary Table S1: Mean herbivorous fish biomass (g m^{-2}) by species between management strata and among habitat types. Species are ordered by mean average biomass in the marine reserve. Standard deviation (s.d.) provided in parenthesis.

Taxa	Herbivore Functional Group	Reserve				Open			
		Crest	Slope	Flat	**All**	Crest	Slope	Flat	**All**
Scarus psittacus	Scraper	24.7 (14.9)	7.49 (9.13)	0.72 (2.63)	**9.75 (13.8)**	9.96 (5.54)	3.52 (3.01)	0.00 (0.01)	**4.39 (5.60)**
Chlorurus spilurus	Scraper	8.86 (4.78)	15.0 (9.50)	0.01 (0.04)	**7.01 (8.47)**	8.08 (5.89)	7.26 (6.90)	0.01 (0.03)	**4.73 (6.16)**
Chlorurus perspicillatus	Scraper	2.43 (4.39)	14.6 (13.4)	0.47 (1.74)	**5.19 (9.68)**	1.57 (2.99)	4.39 (6.92)	0.00 (0.00)	**1.71 (4.25)**
Acanthurus xanthopterus	Grazer	0.01 (0.03)	12.2 (35.0)	0.37 (0.83)	**3.73 (19.1)**	0.00 (0.00)	0.06 (0.16)	0.00 (0.00)	**0.02 (0.08)**
Acanthurus triostegus	Grazer	7.49 (5.36)	1.32 (1.48)	0.68 (1.96)	**2.87 (4.38)**	2.54 (1.73)	0.39 (0.79)	0.00 (0.00)	**0.98 (1.58)**
Acanthurus blochii	Grazer	1.55 (1.49)	3.36 (2.16)	0.19 (0.48)	**1.52 (1.94)**	0.45 (0.57)	0.60 (0.51)	0.00 (0.00)	**0.32 (0.49)**
Acanthurus dussumieri	Grazer	0.30 (0.42)	1.71 (2.02)	0.01 (0.05)	**0.60 (1.31)**	0.01 (0.02)	0.15 (0.20)	0.00 (0.00)	**0.04 (0.12)**
Zebrasoma veliferum	Grazer	0.68 (0.56)	0.67 (0.40)	0.12 (0.29)	**0.45 (0.49)**	0.30 (0.33)	0.34 (0.23)	0.00 (0.00)	**0.19 (0.27)**
Zebrasoma flavescens	Grazer	0.44 (0.51)	0.94 (0.84)	0.02 (0.05)	**0.41 (0.64)**	0.19 (0.37)	0.78 (1.52)	0.00 (0.00)	**0.27 (0.85)**
Naso unicornis	Browser	0.02 (0.06)	1.32 (3.24)	0.00 (0.00)	**0.39 (1.80)**	0.04 (0.15)	0.31 (1.11)	0.00 (0.02)	**0.10 (0.58)**
Naso brevirostris	Browser	0.00 (0.00)	1.24 (3.50)	0.00 (0.00)	**0.36 (1.91)**	0.01 (0.02)	0.00 (0.00)	0.00 (0.00)	**0.00 (0.01)**
Scarus spp.	Scraper	0.04 (0.10)	0.65 (1.14)	0.00 (0.01)	**0.20 (0.66)**	0.52 (0.92)	0.34 (1.13)	0.00 (0.00)	**0.27 (0.81)**
Acanthurus leucopareius	Grazer	0.00 (0.00)	0.67 (1.52)	0.00 (0.00)	**0.20 (0.85)**	0.08 (0.22)	0.14 (0.26)	0.00 (0.00)	**0.07 (0.19)**
Scarus dubius	Scraper	0.07 (0.15)	0.18 (0.38)	0.00 (0.00)	**0.07 (0.23)**	0.11 (0.23)	0.42 (0.96)	0.00 (0.00)	**0.15 (0.53)**
Acanthurus nigrofuscus	Grazer	0.16 (0.32)	0.04 (0.07)	0.00 (0.00)	**0.06 (0.18)**	0.04 (0.12)	0.04 (0.06)	0.00 (0.00)	**0.02 (0.08)**
Scarus rubroviolaceus	Scraper	0.01 (0.02)	0.04 (0.11)	0.00 (0.00)	**0.01 (0.06)**	0.08 (0.16)	0.09 (0.32)	0.00 (0.02)	**0.05 (0.19)**

Supplementary Table S2: Summary of herbivorous fish tagged in the marine reserve and detected for at least one year, showing number of individuals tagged for each species, mean size (TL), days at large (from release date to last detection), total days detected, and percent of days detected in the reserve vs. open areas. Standard deviation (s.d.su) provided in parenthesis.

**Species tagged**	**N**	**mean size (cm)**	**days at large**	**days detected**	**% days detected in reserve**
Naso unicornis	10	30 (7)	375 (36)	199 (93)	88 (15)
Calotomus carolinus	3	47 (3)	399 (12)	88 (49)	99 (1)
Cholorurus perspicillatus	2	29 (2)	399 (11)	66 (73)	100 (0)
All combined	15	33 (8)	383 (32)	159 (99)	92 (14)

Supplementary Table S3: Full diet results showing number of OTUs attributed to each algal species for each fish species. Algal and fish species are in decreasing order by total OTUs.

	Acanthurus triostegus	*Naso brevirostris*	*Acanthurus xanthopterus*	*Naso unicornis*	*Acanthurus blochii*	*Chlorurus perspicillatus*	*Calotomus carolinus*	**Total**
Asparagopsis taxiformis	2797	7	1308	70	34	32	86	4334
Gracilaria salicornia	495	1882	0	733	130	6	1	3247
*Prasinophyceae sp.**	33	1126	585	60	67	6	62	1939
Acanthophora spicifera	1146	0	0	36	0	1	0	1183
Polysiphonia sp.	868	265	0	10	0	2	0	1145
Kappaphycus sp.	0	9	0	811	2	3	1	826
Taenioma perpusillum	581	0	0	0	0	0	0	581
Hypnea sp.	436	6	0	23	0	0	0	465
Colpomenia sinuosa	266	0	0	0	0	0	0	266
Asteronema breviarticulatum	1	0	1	0	0	220	0	222
Gayliella sp.	214	1	0	0	0	0	0	215
Sphacelaria tribuloides	167	0	0	27	0	0	0	194
Ectocarpales sp.	145	0	0	11	4	0	28	188
Anotrichium tenue	178	0	0	0	0	0	0	178
Anotrichium sp.	125	0	0	0	0	0	0	125
Perikladosporon percurrens	71	1	2	0	0	0	0	74
Rhodolachne decussata	67	0	2	0	0	0	0	69
Peyssonnelia inamoena	52	3	0	0	0	0	0	55
Hypnea valentiae	45	0	0	0	0	0	0	45
Gelidiopsis scoparia	0	0	0	0	38	0	0	38
Gelidiella sp.	33	0	0	0	0	0	0	33
Monosporus indicus	1	1	0	21	9	0	0	32
Ceramiales sp.	24	7	0	0	0	0	0	31
Rhodachlya sp.	21	0	0	0	1	0	0	22
Heterosiphonia crispella	15	4	0	0	0	0	0	19
Wrangelia elegantissima	1	7	6	1	2	1	0	18
Chondrophycus cartilagineus	10	0	0	0	0	0	0	10
Lithophyllum kotschyanum	10	0	0	0	0	0	0	10
Herposiphonia sp.	9	0	0	0	0	0	0	9
Dasya iridescens	7	0	0	0	0	0	0	7
Peyssonnelia rubra	0	3	0	3	0	0	0	6
Rhodymeniales sp.	6	0	0	0	0	0	0	6
Aglaothamnion sp.	4	0	1	0	0	0	0	5
Euptilocladia magruderi	5	0	0	0	0	0	0	5
Spirocladia barodensis	0	0	1	3	1	0	0	5
**Total OTUs**	7833	3322	1906	1809	288	271	178	15607
**Species count**	31	5	13	8	14	8	10	35

Details of diet analysis:

We carried out Polymerase Chain Reactions (PCRs) to simultaneously amplify fragments of the 23S plastid rRNA barcoding gene using as template the DNA of virtually all the algae present in each sample (one sample per fish species). We used modified UPA universal primers [p23SrVf1 (5'- GGA CAG AAA GAC CCT ATG AA- 3') and p23SrVr1 (5'- TCA GCCTGT TAT CCC TAG AG – 3')] that have been shown to provide good taxonomic resolution across a wide range of algal diversity [1]. Forward primers were modified in the 5'ends with a 6 bp-indexing tag that was different for each fish species. Reactions were carried out in a 50 µl volume containing ~20 ng of template DNA, 0.1 µM of each primer, and 25 µl of BioMix Red™ (Bioline Inc., Springfield, NJ, USA) in deionized water. Cycling conditions were: an initial denaturation step of 94 °C for 2 min, 35 cycles of denaturation (94 °C for 20 s), annealing (55 °C for 30 s) and extension (72 °C for 30 s), and a final extension step of 72 °C for 10 min. PCR products were purified using QIAQuick PCR purification protocol (Qiagen, Chatsworth, CA) and quantified as described above. Finally, we pooled the purified PCR products, prepared sequencing libraries following Pacific Bioscience's Circular Consensus Sequencing protocol at the Yale Center for Genome Analyses, and sequenced in two flow cells in a Pacific Bioscience RS system.

The bioinformatic analyses were conducted with the help of scripts of the QIIME *vers.* 1.6 pipeline (www.qiime.org[2]. We first quality filtered and demultiplexed the reads based on the 6-bp tag, so that all subsequent analyses were carried out independently for each fish species. The next step consisted of clustering sequences by sequence similarity into Operational Taxonomic Units (OTUs) that were then assumed to represent evolutionarily distinct lineages. To determine the optimal threshold of sequence similarity we studied the value at which the number of OTUs became stable (i.e. where intra- and inter-specific variability minimally affected diversity estimations) and calculated diversity estimates at different sequence similarity thresholds in a dataset of known species composition. Using this approach, we determined that 97% sequence similarity was the optimal threshold, which we then applied for all subsequent analyses.

1. Sherwood, A. R. & Presting, G. G. Universal primers amplify a 23s rdna plastid marker in eukaryotic algae and cyanobacteria1. *J. Phycol.* **43,** 605–608 (2007).
2. Caporaso, J. G. *et al.* QIIME allows analysis of high-throughput community sequencing data. *Nat. Methods* **7,** 335–336 (2010).
